# Ribavirin Treatment Failure-Associated Mutation, Y1320H, in the RNA-Dependent RNA Polymerase of Genotype 3 Hepatitis E Virus (HEV) Enhances Virus Replication in a Rabbit HEV Infection Model

**DOI:** 10.1128/mbio.03372-22

**Published:** 2023-02-21

**Authors:** Bo Wang, Hassan M. Mahsoub, Wen Li, C. Lynn Heffron, Debin Tian, Anna M. Hassebroek, Tanya LeRoith, Xiang-Jin Meng

**Affiliations:** a Department of Biomedical Sciences and Pathobiology, Virginia-Maryland College of Veterinary Medicine, Virginia Polytechnic Institute and State University, Blacksburg, Virginia, USA; b Center for Emerging, Zoonotic and Arthropod-borne Pathogens, Fralin Life Sciences Institute, Virginia Polytechnic Institute and State University, Blacksburg, Virginia, USA; Indiana University Bloomington

**Keywords:** chronic hepatitis E (CHE), hepatitis E virus (HEV), genotype 3 HEV (HEV-3), rabbit HEV-3ra, ribavirin (RBV) treatment failure-associated mutations

## Abstract

Chronic hepatitis E virus (HEV) infection has become a significant clinical problem that requires treatment in immunocompromised individuals. In the absence of an HEV-specific antiviral, ribavirin (RBV) has been used off-label, but treatment failure may occur due to mutations in the viral RNA-dependent RNA polymerase (RdRp), including Y1320H, K1383N, and G1634R. Chronic hepatitis E is mostly caused by zoonotic genotype 3 HEV (HEV-3), and HEV variants from rabbits (HEV-3ra) are closely related to human HEV-3. Here, we explored whether HEV-3ra, along with its cognate host, can serve as a model to study RBV treatment failure-associated mutations observed in human HEV-3-infected patients. By utilizing the HEV-3ra infectious clone and indicator replicon, we generated multiple single mutants (Y1320H, K1383N, K1634G, and K1634R) and a double mutant (Y1320H/K1383N) and assessed the role of mutations on replication and antiviral activity of HEV-3ra in cell culture. Furthermore, we also compared the replication of the Y1320H mutant with the wild-type HEV-3ra in experimentally infected rabbits. Our *in vitro* analyses revealed that the effects of these mutations on rabbit HEV-3ra are altogether highly consistent with those on human HEV-3. Importantly, we found that the Y1320H enhances virus replication during the acute stage of HEV-3ra infection in rabbits, which corroborated our *in vitro* results showing an enhanced viral replication of Y1320H. Taken together, our data suggest that HEV-3ra and its cognate host is a useful and relevant naturally occurring homologous animal model to study the clinical relevance of antiviral-resistant mutations observed in human HEV-3 chronically-infected patients.

## INTRODUCTION

Hepatitis E virus (HEV) infection is one of the most common causes of acute viral hepatitis, with an estimated 20 million HEV infections each year globally leading to 44,000 deaths (1, 2). HEV primarily transmits to humans via the fecal-oral route by drinking contaminated water in developing countries and consuming raw or undercooked animal meat products in industrialized nations (3). HEV is classified in the family *Hepeviridae* (4, 5). Four major HEV genotypes (HEV-1 to -4) within the species *Paslahepevirus balayani* infect humans. HEV-1 and -2 exclusively infect humans; HEV-3 and -4 infect humans and several other animals, such as pigs (6). Unlike other human hepatotropic viruses, HEV has more than a dozen animal reservoirs, and HEV strains from pigs, rabbits, deer, camels, and rats can cross species barriers and cause zoonotic infection in humans (7, 8). Virions of HEV in feces are nonenveloped, but virus particles in circulating blood and supernatant of cell cultures are quasi-enveloped associated with a lipid membrane (9). HEV infection usually develops a self-limiting acute viral hepatitis; however, the majority of zoonotic HEV-3 infections in immunocompromised individuals, such as solid-organ transplant recipients, can progress into chronicity, leading to liver fibrosis and cirrhosis (10, 11), which has now become a significant clinical problem. In addition, HEV-3 infection is also associated with a wide range of extrahepatic manifestations, such as neurological and renal injuries (12). Both chronic hepatitis E (CHE) and neurological sequelae require antiviral treatment.

Ribavirin (RBV) is a guanosine analogue used to exert an antiviral action by causing lethal mutations and suppressing viral RNA-dependent RNA polymerase (RdRp) (13, 14). Although an HEV-specific antiviral is still lacking, the effectiveness of the broad-spectrum antiviral RBV monotherapy to treat CHE has been confirmed (15). Nonetheless, RBV treatment can cause numerous side effects, such as dose-dependent anemia. More importantly, RBV induces viral mutagenesis and increases HEV heterogeneity, and treatment failure may occur due to the emergence of antiviral-resistant variants (16–18). Several amino acid changes in the RdRp region of the HEV genome have been reportedly associated with RBV treatment failure (19–23). *In vitro* studies have demonstrated that the Y1320H and G1634R mutations enhance HEV-3 replication efficiency, whereas K1383N mutation increases RBV susceptibility (21). However, the precise roles of these described mutations in HEV pathogenesis and clinical relevance need to be verified in a suitable animal model.

Rabbit HEV homologs were first reported in 2009 in China (24). Subsequently, a large number of rabbit HEV variants were detected in several other countries, including the United States, France, Germany, Italy, the Netherlands, Canada, Australia, and South Korea, indicating that rabbits are the natural reservoirs of rabbit HEV (25, 26). Since HEV strains from rabbits have a high level of genomic sequence homology with human and swine HEV-3 and are phylogenetically clustered with HEV-3 despite forming a distinct group, the International Committee on the Taxonomy of Viruses (ICTV) *Hepeviridae* Study Group has provisionally assigned the rabbit HEV homologs to HEV-3, named HEV-3ra (6). Indeed, HEV-3ra from rabbits and HEV-3 from humans share many similarities in pathogenesis, transmission pattern, and clinical course. Like human HEV-3, ample evidence has documented that HEV-3ra induces persistent infection in rabbits (27–31). HEV-3ra can infect pigs and cynomolgus monkeys under experimental conditions and propagate in multiple cultured human cells, suggesting potential cross-species transmission (32–34). Conversely, swine HEV can experimentally infect rabbits and produce brain and kidney injuries (35–38). Of note, numerous cases of zoonotic HEV-3ra infections in humans have been reported in several European countries, including France, Switzerland, and Ireland, and the majority of HEV-3ra infections in humans have occurred in immunosuppressed patients, demonstrating the zoonotic risk of HEV-3ra (39–41).

Rabbits have been used as an appropriate animal model for the evaluation of antivirals (e.g., sofosbuvir) and vaccines (e.g., HEV239) against HEV infection (42–47). Strikingly, in analogy to HEV-1-associated high mortality during pregnancy, HEV-3ra reportedly induces adverse fetal outcomes as well as severe liver injury in pregnant rabbits (45, 48), suggesting that rabbits could be used as a promising animal model to study the potential mechanisms of HEV-1-associated fulminant hepatic failure in pregnant women (49, 50).

Taken together, given the close evolutionary relationships between human HEV-3 and rabbit HEV-3ra, rabbits along with HEV-3ra can serve as a model to explore the mechanisms of chronic and extrahepatic HEV-3 infections (38, 51). In this study, we aimed to determine the effect of three clinically reported RBV treatment failure-associated HEV-3 RdRp mutations (Y1320H, K1383N, and G1634R) using HEV-3ra and rabbits as a model. Our comprehensive *in silico* analyses demonstrated the close evolutionary relationships between rabbit HEV-3ra and human HEV-3. We further performed comparative mutational and antiviral analyses of distinct HEV-3ra mutants using reverse genetic and indicator replicon systems *in vitro*. The mutation (Y1320H) with the most significant enhancement of HEV-3ra replication *in vitro* was used to experimentally infect rabbits, which exhibited a replication-enhanced phenotype during the acute stage of virus infection in Y1320H-infected rabbits. Our data provide an experimental rationale for using HEV-3ra and its cognate host as a surrogate model to study the potential function of RBV treatment failure-associated HEV-3 RdRp mutations identified in CHE patients.

## RESULTS

### Rabbit HEV-3ra is evolutionarily closely related to human HEV-3.

**FIG 1:**
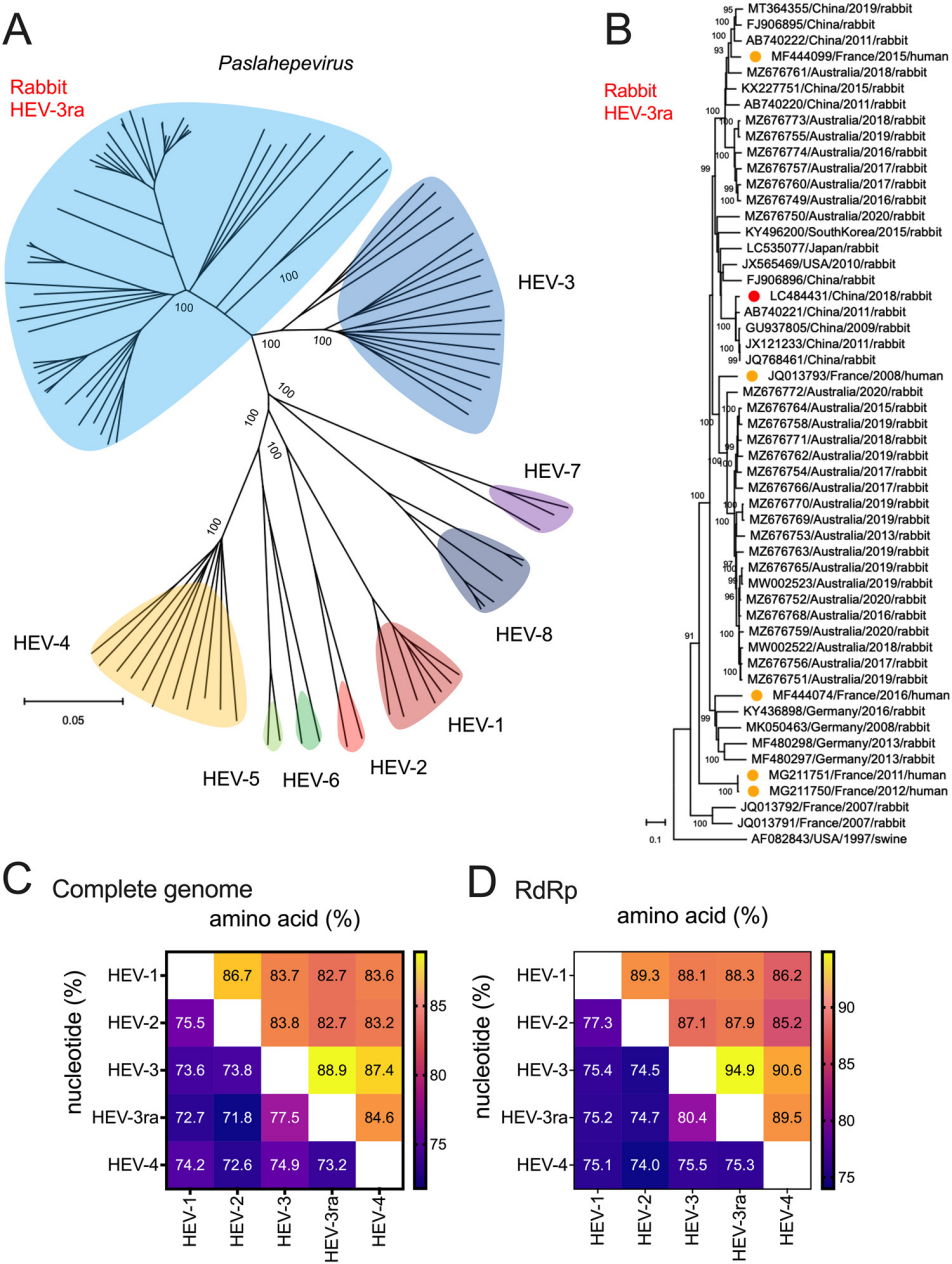
Phylogeny of rabbit HEV-3ra and sequence identities between rabbit HEV-3ra and four major HEV genotypes. (A) A neighbor-joining tree of maximum likelihood distances was generated based on HEV genomic sequences of representative members within the genus *Paslahepevirus*. Clusters of eight distinct HEV genotypes and the rabbit HEV-3ra were highlighted with different colors. (B) A maximum-likelihood tree was generated based on 52 genomic sequences of rabbit HEV-3ra. The HEV-3 reference Meng strain (GenBank accession no. AF082843) served as an outgroup. Virus designations include GenBank accession number, country origin, and host. HEV-3ra sequences used in this study and those detected in humans are highlighted with red- and orange-filled circles, respectively. Evolutionary analyses were conducted in molecular evolutionary genetics analysis 11 (MEGA 11) based on the multiple sequence alignment in Geneious Prime. General time reversible (GTR) + gamma distributed (G) + invariable sites (I) nucleotide substitution model with the lowest Bayesian information criterion (BIC) score was selected based on the find best-fit substitution model (ML) in MEGA 11. Bootstrap values (>90%) are presented at specific nodes. Scale bars indicate the estimated number of nucleotide substitutions per site. (C) Comparisons of nucleotide sequences of complete genomes and amino acid sequences of concatenated ORF1 and ORF2 among four major HEV genotypes and HEV-3ra. (D) Comparisons of nucleotide and amino acid sequences of RNA-dependent RNA polymerase (RdRp) among four major HEV genotypes and HEV-3ra. Representative viral genomes of eight HEV genotypes and HEV-3ra were included for analyses.

Thus far, eight major HEV genotypes have been assigned to the species *balayani* of the genus *Paslahepevirus.* Each genotype has a distinct host range and infection pattern (4, 6). According to the consensus proposal from the ICTV *Hepeviridae* Study Group, the rabbit HEV strains were assigned a subtype within HEV-3, designated HEV-3ra (6). Phylogenetically, representative members of eight HEV genotypes of the genus *Paslahepevirus* clustered separately, and the HEV-3ra strains formed a sister clade to the HEV-3 (Fig. 1A). Notably, the HEV-3ra clearly showed an even higher genetic diversity than HEV-3, let alone other HEV genotypes (Fig. 1A), reflecting an extremely long-term virus-host evolutionary history of HEV-3ra in rabbits (52). Given the broad host tropism and frequent cross-species transmission of HEV-3 (5, 8), it is therefore speculated that rabbits (order Lagomorpha) accidentally acquired the ancient HEV-3, likely due to the occurrence of intensive animal husbandry farming (53). A total of 52 genomic sequences of HEV-3ra are currently available in the GenBank database (retrieved as of October 2022), which were derived from multiple countries worldwide, with the vast majority from Australia. Although three independent studies from France, Switzerland, and Ireland have reported numerous cases of zoonotic HEV-3ra infection in humans, complete HEV genomes in only five French patients were reported. We found that these five strains dispersed in the rabbit HEV-3ra phylogenetic tree (Fig. 1B), suggesting that other HEV-3ra strains are likely zoonotic as well. The HEV-3ra LR strain (GenBank accession no. LC484431) located within the rabbit HEV-3ra cluster was isolated from Inner Mongolia, China, and its reverse genetic system was recently developed (Fig. 1B) (27).

We further compared the sequence identities between HEV-3ra and four major human-infecting HEV genotypes (HEV-1 to -4). As anticipated, HEV-3ra has the highest genomic sequence homology to human HEV-3 at both the nucleotide (77.5%) and amino acid (88.9%) levels (Fig. 1C). Sequence comparisons of the RdRp region of the HEV genome demonstrated that HEV-3ra shared sequence identity of 80.4% at the nucleotide level and 94.9% at the amino acid level, much higher than that of other genotypes (Fig. 1D). Therefore, our phylogenetic and sequence analyses revealed that the rabbit HEV-3ra is evolutionarily closely related to human HEV-3. The high genomic similarity between rabbit HEV-3ra and human HEV-3 in the RdRp region, as demonstrated in our *in silico* analyses here, suggests that HEV-3ra would be a valuable surrogate model for studying the effect of RdRp mutations on human HEV-3 replication and pathogenesis.

### Mapping the three RBV treatment failure-associated human HEV-3 RdRp mutations (Y1320H, K1383N, and G1634R) to the HEV-3ra genome and epidemiological prevalence of three mutations among 8 HEV genotypes and HEV-3ra.

**FIG 2:**
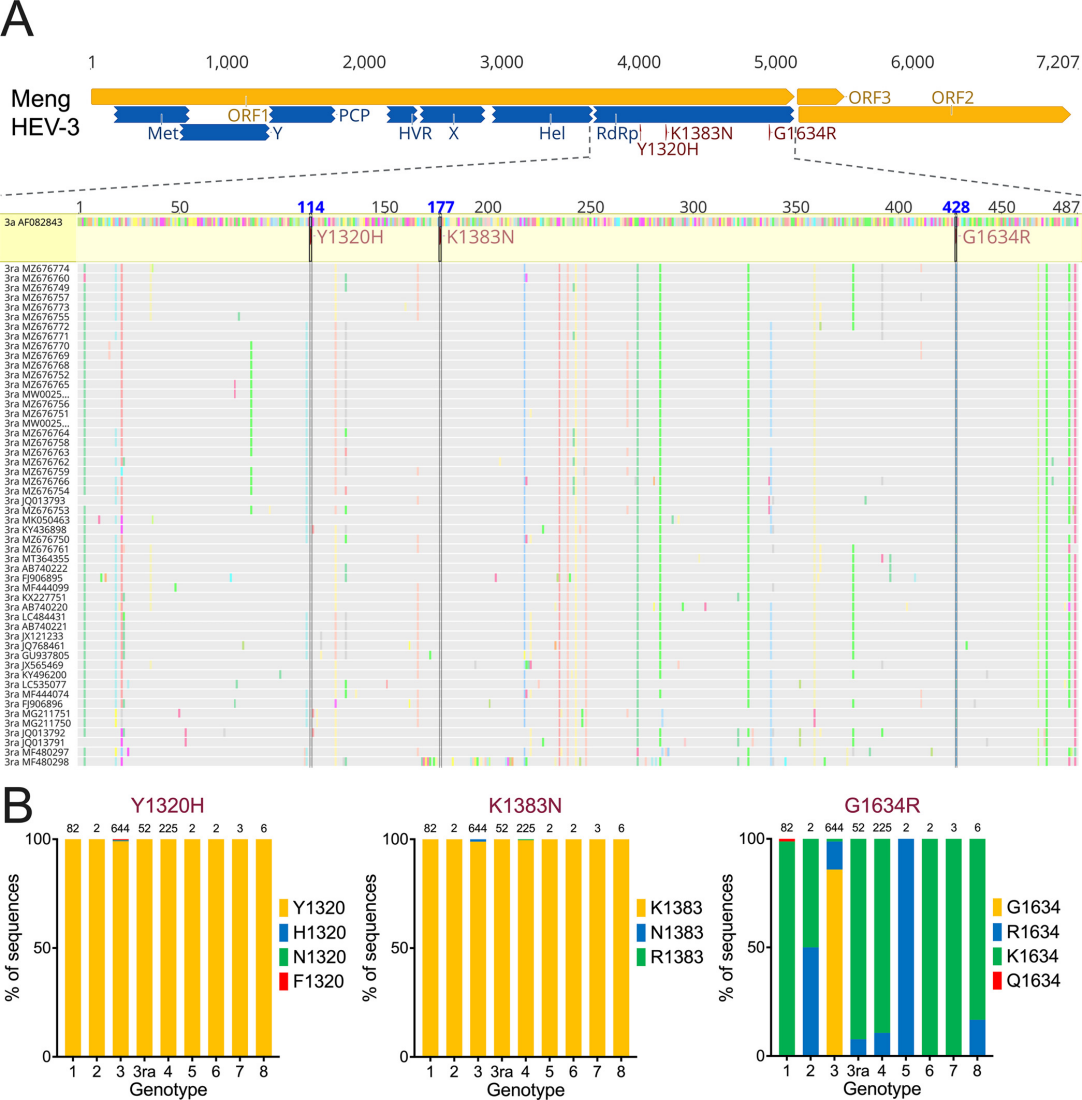
Locations of three RBV treatment failure-associated mutations in HEV-3 RdRp and epidemiological prevalence of the mutations at different amino acid positions among the eight HEV genotypes and HEV-3ra. (A) Three mutations in the RdRp (Y1320H, K1383N, and G1634R) that are associated with RBV treatment failure reported in a clinical CHE case are shown in the HEV-3 reference Meng strain (GenBank accession no. AF082843). The putative functional domains within ORF1 are depicted: Met, methyltransferase; Y, Y domain; PCP, papain-like cysteine protease; HVR, hypervariable region; PPR, poly proline region; X, macro domain; Hel, helicase; RdRp, RNA-dependent RNA polymerase. The viral genome in nucleotide bases is shown on the top. The RdRp region within ORF1 of 52 rabbit HEV-3ra genomes is aligned with the HEV-3 Meng strain, and the locations of these three mutations are mapped and highlighted in the genomes of HEV-3ra. (B) Epidemiological prevalence of the three RBV treatment failure-associated HEV-3 RdRp mutations at different amino acid positions among eight HEV genotypes and HEV-3ra. HEV full-length genomes were retrieved (as of October 2022) from the GenBank database and aligned for comprehensive sequence analyses. The numbers of viral genomes analyzed for each genotype are 82 for HEV-1, 2 for HEV-2, 644 for HEV-3, 52 for HEV-3ra, 225 for HEV-4, 2 for HEV-5, 2 for HEV-6, 3 for HEV-7, and 6 for HEV-8. The amino acid residues of each mutation are presented in different colors.

Recently, multiple HEV-3 RdRp mutations, including Y1320H, K1383N, D1384G, K1398R, V1439I, Y1587F, and G1634R, have been reportedly associated with RBV treatment failure in CHE patients in several European countries (18, 20, 54); among them, the Y1320H, K1383N, and G1634R were identified in a patient experiencing RBV monotherapy treatment failure and have therefore been further studied *in vitro* using the human HEV-3 infectious clone Kernow-C1 p6 (GenBank accession no. JQ679013) and indicator replicon p6Gluc (21), which revealed the roles of enhanced viral replication for Y1320H and G1634R and the increased RBV sensitivity for K1383N. However, the mechanisms of these mutations on RBV sensitivity and viral replication remain poorly understood since *in vivo* experimental confirmation in a suitable and relevant animal model is still lacking. To investigate the potential impact of RBV treatment failure-associated HEV-3 RdRp mutations on rabbit HEV-3ra, we performed sequence alignment of the HEV-3 reference Meng strain (GenBank accession no. AF082843) and 52 strains of HEV-3ra and successfully mapped the three RBV treatment failure-associated HEV-3 RdRp mutations to HEV-3ra (Fig. 2A). We found that the amino acid residues at positions 1320 and 1383 of HEV-3ra are Y (Tyr) and K (Lys), respectively, which are identical to those of HEV-3; however, the primary amino acid residues at position 1634 of rabbit HEV-3ra are K (Lys) and R (Arg) but not G (Gly).

To determine the relative epidemiological prevalence of these mutations at their respective positions, we conducted comparative analyses of each of three RBV treatment failure-associated HEV-3 RdRp mutations (Y1320H, K1383N, and G1634R) among eight different HEV genotypes and HEV-3ra (82 genomes for HEV-1, 2 genomes for HEV-2, 644 genomes for HEV-3, 52 genomes for HEV-3ra, 225 genomes for HEV-4, 2 genomes for HEV-5, 2 genomes for HEV-6, 3 genomes for HEV-7, and 6 genomes for HEV-8) (Fig. 2B; see also Table S1 in the supplemental material). We found that the amino acid residues at positions 1320 and 1383 are highly conserved, with a prevalence of nearly 100% among the eight distinct HEV genotypes as well as HEV-3ra. Specifically, in 52 HEV-3ra genomes, the prevalence of Y1320 and K1383 is 100%. In contrast, the amino acid residue at position 1634 is disordered. In HEV-3 genomes, the G1634 is predominant (85.87%, 554/644) followed by R (12.89%, 83/644), but the G1634 rarely exists in other HEV genotypes, including HEV-3ra. Notably, K1634 (92.31%) appears in 48 of 52 HEV-3ra genomes; the remaining four are with R1634 (7.69%). Intriguingly, K1634 seems to be favored in most HEV genotypes, although there are only two viral genomes currently available for HEV-2, -5, and -6, owing to the sampling bias (55).

10.1128/mbio.03372-22.2 TABLE S1 Prevalence of different amino acid residues at positions of the three ribavirin treatment failure-associated HEV-3 RdRp mutations for each of the eight HEV genotypes and rabbit HEV-3ra within the genus *Paslahepevirus*. Download Table S1, DOCX file, 0.02 MB. Copyright © 2023 Wang et al. 2023 Wang et al. https://creativecommons.org/licenses/by/4.0/ This content is distributed under the terms of the Creative Commons Attribution 4.0 International license.

### Y1320H mutation significantly enhances HEV-3ra (LR) replication in cell culture.

**FIG 3:**
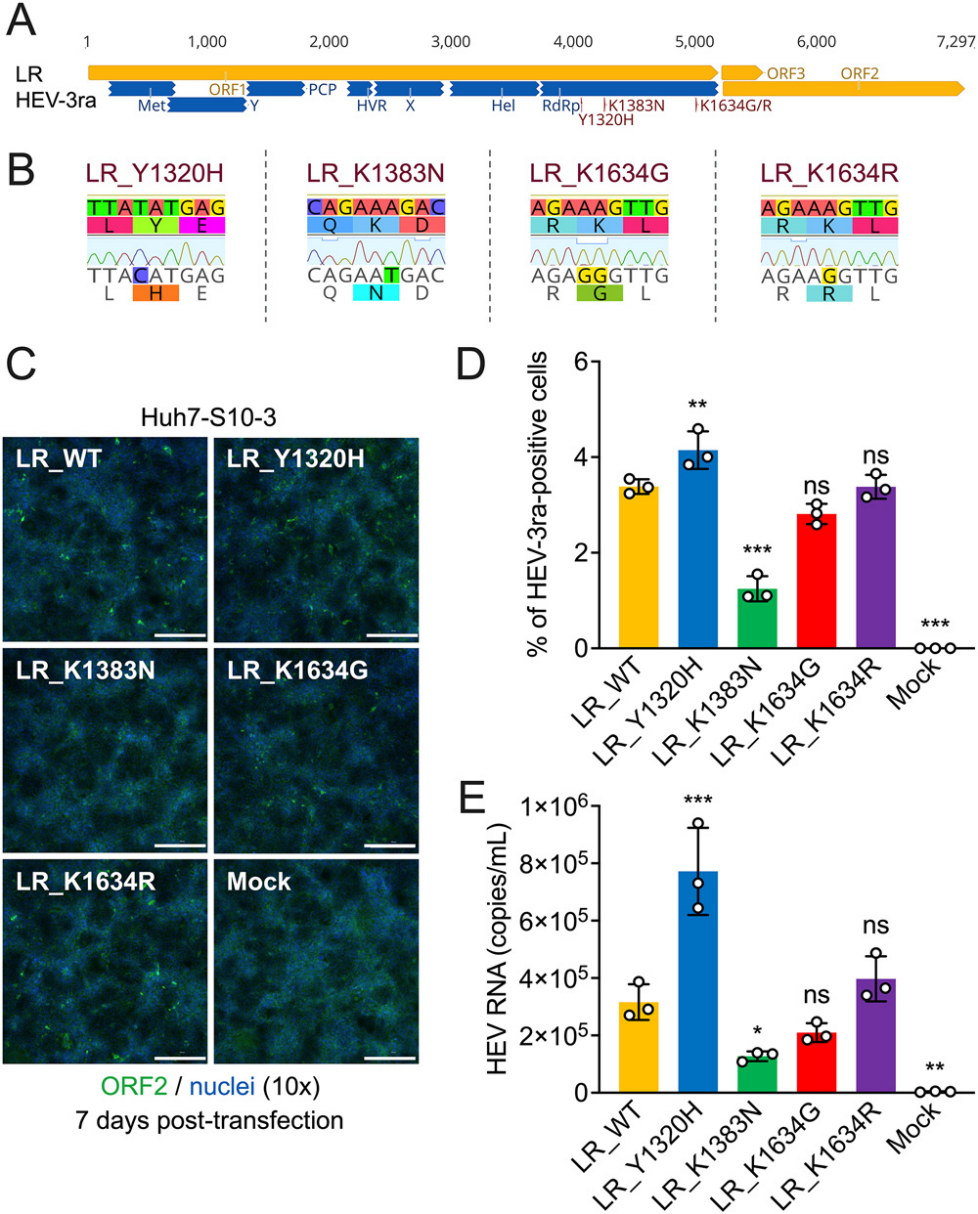
Effect of RBV treatment failure-associated individual HEV-3 RdRp mutations on *in vitro* replication efficiency of HEV-3ra. (A) Schematic representation of the rabbit HEV-3ra infectious clone LR. The putative functional domains within ORF1 are depicted: Met, methyltransferase; Y, Y domain; PCP, papain-like cysteine protease; HVR, hypervariable region; PPR, poly proline region; X, macro domain; Hel, helicase; RdRp, RNA-dependent RNA polymerase. The three selected RBV treatment failure-associated HEV-3 RdRp mutations (Y1320H, K1383N, and K1634G/R) are indicated. The genome of the HEV-3ra LR in nucleotide bases is shown on the top. (B) Construction of the following four HEV-3ra LR-derived mutants: Y1320H, K1383N, K1634G, and K1634R. Chromatograms of nucleotide and amino acid substitutions compared with the parental wild-type LR strain are highlighted. (C) Representative immunofluorescence staining of HEV ORF2-positive foci of Huh7-S10-3 cells at 7 days posttransfection of HEV-3ra LR wild type and mutants. HEV ORF2-positive foci are shown in green (anti-ORF2 polyclonal antibody raised from rabbit and goat anti-rabbit monoclonal antibody Alexa Fluor 488), and cell nuclei are shown in blue (DAPI) (scale bar, 200 μm). (D) Number of HEV-3ra ORF2-positive cells at 7 days posttransfection of HEV-3ra LR wild type and mutants. (E) HEV RNA copy numbers were quantified by RT-qPCR from the culture supernatant of Huh7-S10-3 cells at 7 days posttransfection with respective HEV-3 LR wild type and mutants. Values represent means plus standard deviations (SDs) (error bars) from independent experiments (*n *= 3). Statistical differences were determined with one-way ANOVA. *, *P *< 0.05; ***, *P *< 0.001; ns, not statistically significant.

Given that the rabbit HEV-3ra is evolutionarily closely related to human HEV-3, to determine the impact of RBV treatment failure-associated HEV-3 RdRp mutations on the replication of rabbit HEV-3ra, we utilized an established reverse genetic system of an HEV-3ra LR strain (GenBank accession no. LC484431) (Fig. 3A), which has been shown to successfully propagate in human hepatoma PLC/PRF/5 cells and induce persistent viral infection in inoculated rabbits (27). Using the HEV-3ra LR infectious clone as the backbone, we first generated four individual LR mutants (LR_Y1320H, LR_K1383N, LR_K1634G, and LR_K1634R), each containing a single amino acid mutation, which corresponds to one of three RBV treatment failure-associated HEV-3 RdRp mutations (21). Because the original amino acid residue in LR at genomic position 1634 is K (Gly), we mutated K1634 to both G1634 and R1634. In each of the four LR mutants, either one or two nucleotides were correctly substituted from wild-type LR infectious clone using site-directed mutagenesis systems (Fig. 3B; see also Table S2 in the supplemental material). *In vitro* capped RNA transcripts from wild-type LR (LR_WT) and each of four LR mutants were transfected to Huh7-S10-3 liver cells, and the transfected cells were stained with the anti-HEV ORF2 antibody at 7 days posttransfection (dpt). HEV-3ra-positive foci were observed microscopically in LR_WT, LR_Y1320H, LR_K1634G, and LR_K1634R. Remarkably, there were obviously more HEV-3ra-positive foci in LR_Y1320H than in LR_WT (Fig. 3C). The HEV-3ra-positive cells in the immunofluorescence assays were quantified, and the results showed that there were significantly more HEV-3ra-positive foci in LR_Y1320H and significantly fewer positive foci in LR_K1383N. In contrast, there was no significant difference in HEV-3ra-positive foci in LR_K1634G or LR_K1634R compared with that in LR_WT (Fig. 3D). Similar results were also obtained from the quantification of the amounts of virus in the media of transfected cells by measuring viral RNA loads with an HEV-specific reverse transcription-quantitative PCR (RT-qPCR) (56). We found that the LR_Y1320H replicated and/or assembled more efficiently than LR_WT, while LR_K1383N significantly impaired the virus replication. Although neither LR_K1634G nor LR_K1634R significantly affected virus replication compared to the LR_WT, the LR_K1634G replicated at a reduced level and the LR_K1634R at an enhanced level (Fig. 3E).

10.1128/mbio.03372-22.3 TABLE S2 Primers used for rabbit HEV-3ra LR strain genomic sequencing, construction of viral mutants, and generation of LRGluc indicator replicon. Download Table S2, DOCX file, 0.02 MB. Copyright © 2023 Wang et al. 2023 Wang et al. https://creativecommons.org/licenses/by/4.0/ This content is distributed under the terms of the Creative Commons Attribution 4.0 International license.

**FIG 4:**
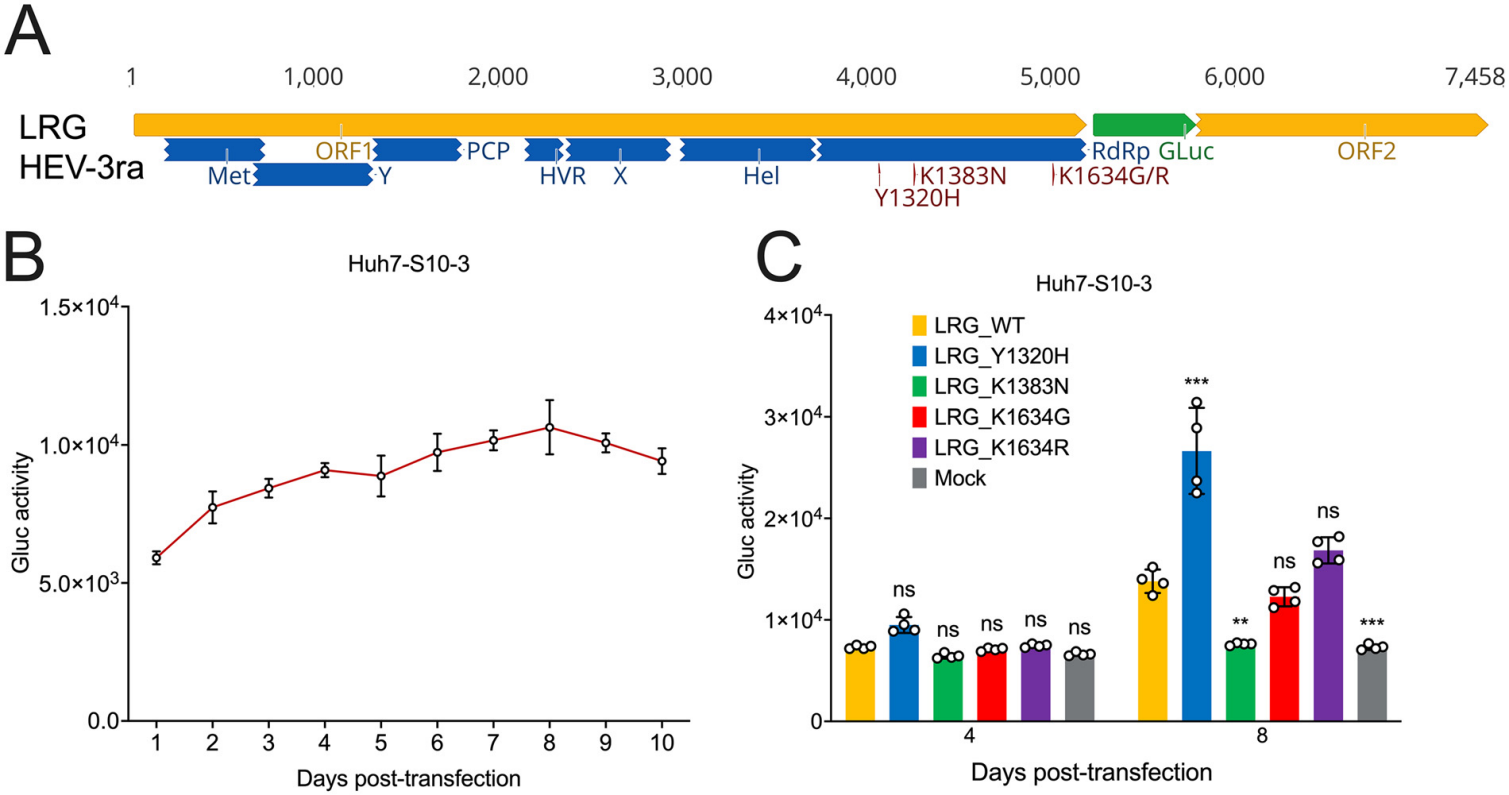
Construction of rabbit HEV-3ra indicator replicon LRG and comparative analyses of replication efficiency of LRG wild type and mutants containing each single RdRp mutation. (A) A schematic representation of the HEV-3ra indicator replicon LRG. The putative functional domains within ORF1 are depicted as follows: Met, methyltransferase; Y, Y domain; PCP, papain-like cysteine protease; HVR, hypervariable region; PPR, poly proline region; X, macro domain; Hel, helicase; RdRp, RNA-dependent RNA polymerase. The three selected RBV treatment failure-associated RdRp mutations (Y1320H, K1383N, and K1634G/R) are indicated, and the *Gaussia* luciferase (GLuc) gene is indicated in green. The genome of the HEV-3ra LRG in nucleotide bases is shown on the top. (B) Growth kinetics of LRG wild type as measured by Gluc expression activity. Cell culture media of Huh7-S10-3 cells were collected at serial time points posttransfection with LRG wild type, and the Gluc activity was monitored. Values represent means plus SD (error bars) from independent experiments (*n *= 4). (C) Comparative analyses of replication efficiency of LRG wild type and mutants. At 4- and 8-days posttransfection with LRG wild type and mutants, respectively, cell culture media of Huh7-S10-3 cells were harvested, and the Gluc expression activity was measured and compared. Values represent means plus SD (error bars) from independent experiments (*n *= 4). Statistical differences were determined with one-way ANOVA. **, *P* < 0.01; ***, *P* < 0.001; ns, not statistically significant.

Recombinant virus replicons that encode indicator genes provide valuable tools for studying viral replication and sensitivity to small molecule inhibitors (19–21). To ensure the reproducibility of our *in vitro* data from the HEV-3ra infectious clone system, we further developed an HEV-3ra indicator replicon system. Briefly, the N-terminal ORF2 sequence of rabbit HEV-3ra LR infectious clone is replaced by a secreted version of the *Gaussia* luciferase (GLuc) gene (Fig. 4A). Two restriction enzymes, SpeI and EcoNI, and overlapping PCRs were employed to construct the indicator replicon of HEV-3ra LR GLuc (designated LRGluc or LRG; GenBank accession no. OP887158). Restriction digestion analysis of the LRGluc plasmid showed the expected size of DNA fragments (see Fig. S1 in the supplemental material). The daily Gluc activity was measured continuously for 10 days, showing that LRGluc is replication competent in Huh7-S10-3 liver cells, 8 dpt would be sufficient to nearly reach peak luminescence (1.1 × 10^4^ units) (Fig. 4B), and LRGluc is suitable for testing HEV-3ra replication efficiency. Subsequently, we constructed four LRGluc single mutants (LRG_Y1320H, LRG_K1383N, LRG_K1634G, and LRG_K1634R), and the Gluc activity of wild-type LRG (LRG_WT) and LRG mutants was tested at 4 and 8 dpt in the culture supernatant of Huh7-S10-3 liver cells (Fig. 4C). At 8 dpt, compared with that of LRG_WT, the LRG_Y1320H demonstrated significantly enhanced viral replication efficiency with a 1.9-fold increase. On the contrary, the LRG_K1383N significantly decreased viral replication to a level of mock transfection. The LRG_K1634G slightly decreased while LRG_K1634R increased viral replication, although the difference is not statistically significant. Therefore, the results derived from the HEV-3ra LR GLuc indicator replicon system are highly consistent with the HEV-3ra LR infectious clone system. Collectively, the impact of three RBV treatment failure-associated HEV-3 RdRp mutations on *in vitro* viral replication using the rabbit HEV-3ra LR model is highly comparable with using the human HEV-3 Kernow-C1 p6 (21), a cell culture-adapted strain (57), indicating that HEV-3ra is a suitable surrogate model for studying the RBV treatment failure-associated HEV-3 RdRp mutations. The major advantage of using rabbit HEV-3ra, instead of human HEV-3 Kernow-C1 p6, is that the potential role of any RBV treatment failure-associated HEV-3 RdRp mutations can be further tested in a small homologous animal host, rabbit, for HEV-3ra.

10.1128/mbio.03372-22.1 FIG S1 An overview of the HEV-3ra indicator replicon LRG construction strategy. Two subgenomic fragments, A1 and A2, of the HEV-3ra infectious clone LR were cloned with restriction sites SpeI and EcoRI, respectively. The GLuc gene was cloned using specific primers. Subsequently, three fragments were amplified with overlapping PCRs, and the N-terminal ORF2 sequence of LR was replaced by the GLuc gene. Agarose gel electrophoresis of PCR products of different LRGluc fragments and digestion of LRGluc with indicated restriction enzymes. Download FIG S1, TIF file, 1.2 MB. Copyright © 2023 Wang et al. 2023 Wang et al. https://creativecommons.org/licenses/by/4.0/ This content is distributed under the terms of the Creative Commons Attribution 4.0 International license.

### Y1320H mutation rescued the K1383N mutation-associated HEV-3ra replication defects, and Y1320H/K1383N double mutant has a markedly higher sensitivity to RBV treatment.

**FIG 5:**
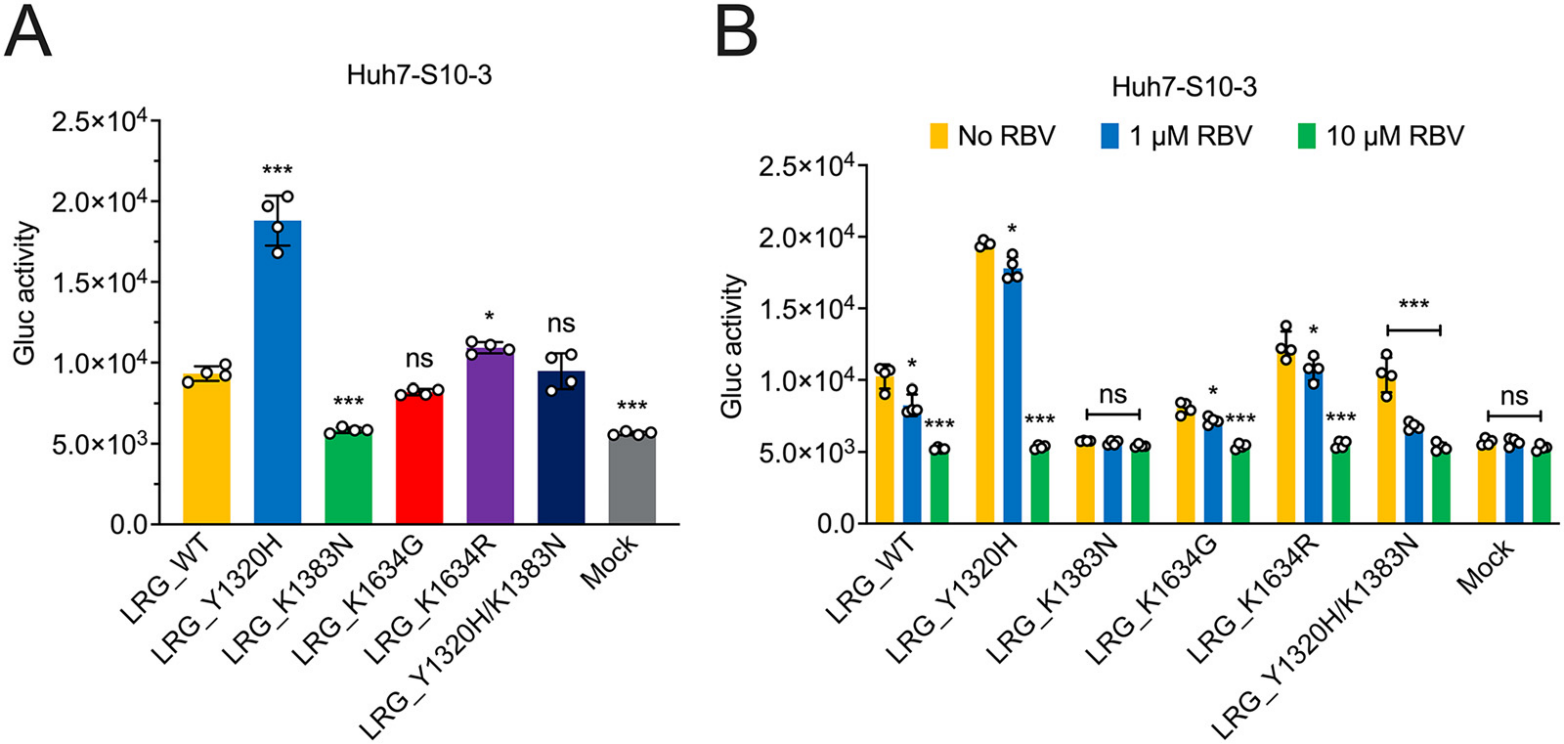
The Y1320H mutation compensated for the K1383N mutation-associated replication defects, and the Y1320H/K1383N double mutant has a markedly higher sensitivity to RBV. (A) Comparative analyses of replication efficiency in Huh7-S10-3 cells of HEV-3ra LRG wild type and mutants containing a single RdRp mutation or a double mutation (Y1320H/K1383N). (B) Comparative analyses of RBV sensitivity to HEV-3ra LRG wild type and mutants. Huh7-S10-3 cells were cultured without RBV or with 1 μM RBV or 10 μM RBV. At 7 days posttransfection with LRG wild-type and mutants, respectively, cell culture media of Huh7-S10-3 cells were collected, and the Gluc expression activity was measured and compared. Values represent means plus SDs (error bars) from independent experiments (*n *= 4). Statistical differences were determined with one-way ANOVA. *, *P *< 0.05; ***, *P *< 0.001; ns, not statistically significant.

It has been reported that Y1320H, G1634R, and the hypervariable region (HVR) insertion can compensate for the K1383N-associated replication defects on the human HEV-3 Kernow-C1 p6 strain (21). To evaluate whether we can reproduce the human HEV-3 p6 results utilizing the rabbit HEV-3ra LR model system, especially given our observation of the highly significant enhancement of Y1320H on HEV-3ra LR replication ability, we further generated the LRG_Y1320H/K1383N double mutant and compared its replication efficiency with LRG_WT and LRG single mutants. We found that the Y1320H rescued the K1383N-associated HEV-3ra replication defects, even to a similar level of LRG_WT (Fig. 5A), which is consistent with the reported impact of Y1320H on human HEV-3.

Additionally, previous *in vitro* studies showed that the K1383N mutation significantly altered viral fitness as well as RBV susceptibility and may play a crucial role in RBV treatment failure in clinical cases (20, 21). To assess the impact of K1383N on the sensitivity of HEV-3ra to RBV, we added 1 μM or 10 μM RBV to Huh7-S10-3 cells transfected with LRG_WT and LRG single and double mutants, and cells without the addition of RBV served as controls. The results from luminescence-based antiviral assays showed that the administration of 1 μM RBV significantly inhibited viral replication, particularly for the LRG_Y1320H/K1383N, and the administration of 10 μM RBV reduced the luminescence activity of LRG_WT and LRG mutant to mock levels (Fig. 5B). Therefore, like human HEV-3, the K1383N mutation also increased the RBV susceptibility for rabbit HEV-3ra. In line with the previous findings regarding the antiviral sensitivity of Y1320H and G1634R mutations using human HEV-3, we also showed that neither Y1320H nor K1634G/R significantly affected RBV susceptibility of HEV-3ra (21). Overall, these data demonstrated that three RBV treatment failure-associated HEV-3 RdRp mutations have a comparable impact on the *in vitro* replication and RBV sensitivity of HEV-3ra, further indicating that HEV-3ra and its cognate host are a good and relevant model system for studying the antiviral resistance and pathogenesis of RBV treatment failure-associated HEV-3 RdRp mutations.

### Physicochemical and structural analyses of RBV treatment failure-associated HEV-3 RdRp mutations.

**FIG 6:**
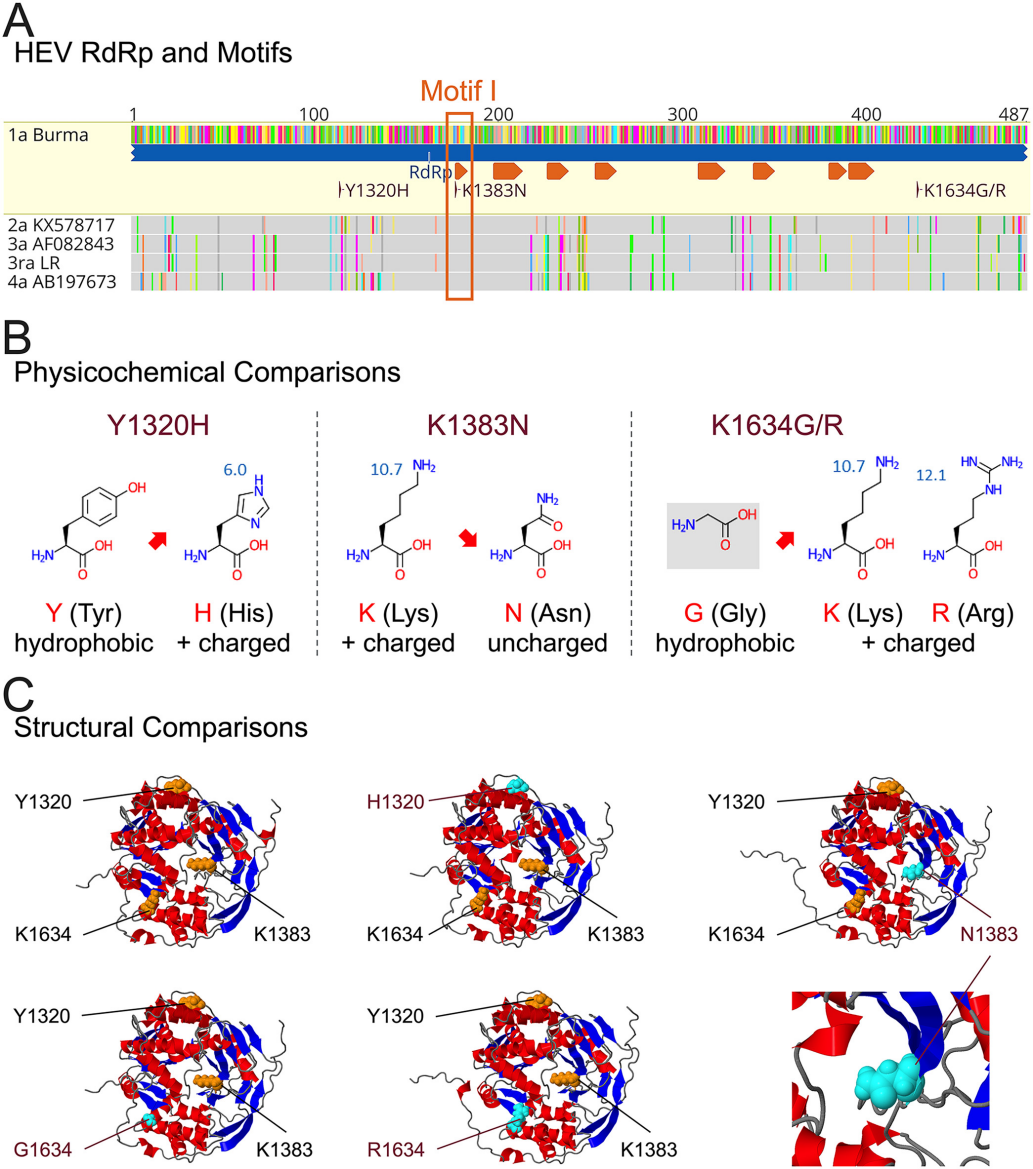
Physicochemical and structural comparisons of RBV treatment failure-associated HEV-3 RdRp mutations. (A) Alignment of the RdRp sequences of four major human-infecting HEV genotypes and rabbit HEV-3ra. The eight motifs in RdRp and three selected RdRp mutations (Y1320H, K1383N, and K1634G/R) are indicated. The RBV treatment failure-associated HEV-3 RdRp mutation K1383N located at motif I is highlighted. Locations of functional domains and motifs within HEV ORF1 are according to the HEV prototype Burma strain (GenBank accession no. M73218) (66). (B) Physicochemical comparisons of the three RBV treatment failure-associated HEV-3 RdRp mutations. The impact of these mutations on *in vitro* replication of HEV-3ra is indicated in red arrows. The physicochemical properties and structures of relevant proteinogenic amino acids in this study are illustrated and adapted from https://commons.wikimedia.org/wiki/File:Proteinogenic_Amino_Acid_Table.png created by Thomas Ryckmans (2022). (C) Structural comparisons of RdRp of wild-type HEV-3ra LR or that with each single RBV treatment failure-associated HEV-3 RdRp mutation. The three-dimensional structures of RdRp are predicted with AlphaFold (68) and visualized and annotated in Geneious Prime software version 2022.2.2. The alpha helices and beta sheets are indicated in red and blue, respectively. The positions of three RBV treatment failure-associated HEV-3 RdRp mutations (114, 177, and 428) with original amino acid residues are shown in orange atoms, and with amino acid changes are highlighted in cyan atoms. The figures only aim to present the locations of the three RBV treatment failure-associated HEV-3 RdRp mutations based on the three-dimensional structure of the RdRp of the HEV-3ra LR strain and do not necessarily represent its true three-dimensional structure.

Based on our experimental results in this study, we showed that each of the three RBV treatment failure-associated HEV-3 RdRp mutations has a distinct impact on the *in vitro* replication of HEV-3ra. In clinical cases, the K1383N occurred in all four patients with RBV treatment failure, but *in vitro* studies showed that this mutation significantly increased RBV sensitivity, which contradicts the clinical phenotype (20, 21). Of note, we found that the K1383N located in motif I of the HEV RdRp region, which is associated with structural conformations, is therefore conserved across eight HEV genotypes and HEV-3ra (Fig. 2B and Fig. 6A). It seems reasonable to speculate that a single nucleotide substitution in this region can essentially affect the secondary structure of HEV RdRp, which may explain the significant reduction of viral replication with the K1383N mutation. In contrast, the Y1320H and K1634G/R are located in nonmotif regions of RdRp, which supposedly tolerate amino acid changes. Intriguingly, although we showed that the 1634 position is relatively heterogenic in HEV genomes, the 1320 position is highly conserved across HEV genotypes (Fig. 2B).

We further conducted physicochemical comparisons of the three RBV treatment failure-associated HEV-3 RdRp mutations and found that the positively charged amino acid residues at positions 1320 and 1634 are preferable to hydrophobic amino acids for HEV-3 *in vitro* replication (Fig. 6B) (19). Specifically, the substitution of hydrophobic amino acid Y (Tyr) to positively charged amino acid H (His) at position 1320 and the substitution of hydrophobic amino acid G (Gly) to positively charged amino acids K (Lys) and R (Arg) at position 1634 increased the replication efficiency of HEV-3 (Fig. 3D and E and Fig. 4C). Additionally, our structural analyses demonstrated that the K1383 is located at the end of a β-sheet, and the introduction of the N1383 mutation transformed the central three-dimensional structure of the RdRp of HEV-3 (Fig. 6C). On the contrary, the H1320 and G/R1634 do not alter the primary structure of the HEV-3. Therefore, it is hypothesized that the K1383N altered the structure of HEV-3 RdRp and eventually led to RBV treatment failure of HEV-3 in CHE patients. Indeed, the selection of K1383N is reversible when RBV therapy stops in clinical cases (20). Nonetheless, the exact underlying mechanisms of HEV-3 RdRp mutations during RBV monotherapy are still to be determined.

### The Y1320H mutant enhances virus replication during the acute stage of infection in HEV-3ra-infected rabbits.

**FIG 7:**
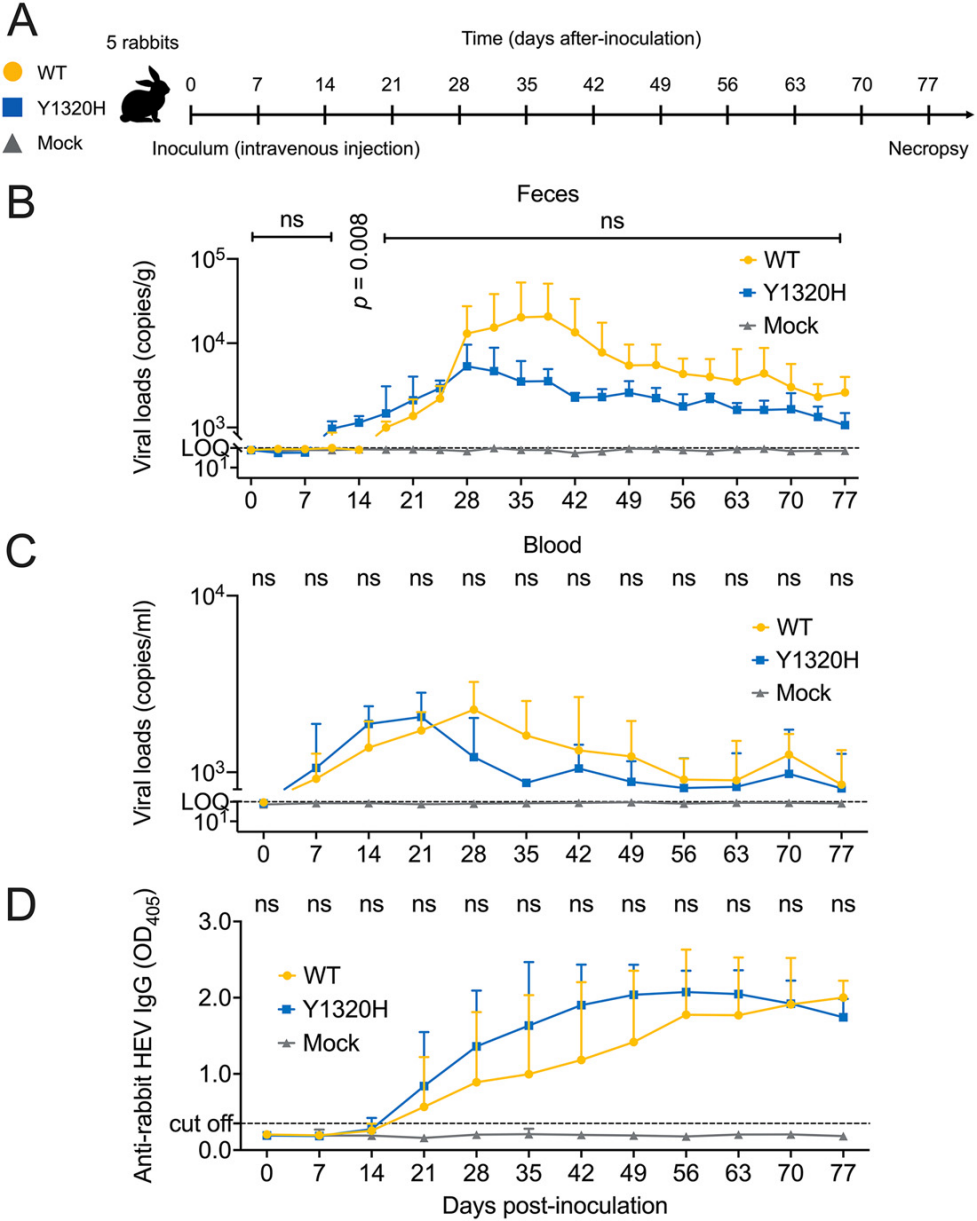
Comparisons of replication and infection dynamics of HEV-3ra LR_WT and LR_Y1320H in rabbits. (A) Illustration of intravenous inoculation via the ear vein of specific-pathogen-free female New Zealand White rabbits with HEV-3ra LR_WT (*n* = 5, yellow, round), LR_Y1320H (*n* = 5, blue, square), and PBS for mock infection (*n* = 5, gray, triangle). Fecal samples were collected from each individual rabbit twice weekly, and serum samples were collected weekly after inoculation. All rabbits were necropsied at 11 weeks (77 days) postinoculation. (B) Kinetics of viral RNA loads during the course of infection in the fecal samples of HEV-3ra LR_WT- and LR_Y1320H-infected rabbits. (C) Kinetics of viral RNA loads during the course of infection in the serum samples of HEV-3ra LR_WT and LR_Y1320H-infected rabbits. (D) Kinetics of anti-HEV-3ra IgG antibody response in rabbits experimentally inoculated with HEV-3ra LR_WT and LR_Y1320H. Seroconversion to anti-HEV IgG antibodies in HEV-3ra LR_WT- and LR_Y1320H-infected rabbits during the course of infection was detected by ELISA with a correct S/N ratio cutoff of 0.35 for positive samples. Values represent means plus SD (error bars) from five animals (*n *= 5) in each group. Statistical differences were determined with a two-sided, unpaired, multiple Student's *t* test without adjustments. *P* value is indicated when less than 0.05; ns, no statistical significance.

It has previously been reported that the Y1320H significantly increased *in vitro* replication of human HEV-3 p6 as well as p6 chimeric constructs with ORF1 sequence from the CHE patient (21). Similarly, as noted above, in this study we also showed that the Y1320H significantly enhanced *in vitro* replication of HEV-3ra LR. To determine the impact of Y1320H mutation on viral replication of HEV-3ra LR *in vivo* using HEV-3ra infection in rabbits as the model, we produced LR_WT and LR_Y1320H infectious virus stocks in transfected Huh7-S10-3 cells and intravenously inoculated female New Zealand White rabbits with LR_WT (*n *= 5), LR_Y1320H (*n *= 5), and phosphate-buffered saline (PBS) (*n *= 5) as negative controls (Fig. 7A).

To monitor the viral infection kinetics in inoculated rabbits, twice-weekly fecal samples and weekly-serum samples from each rabbit in all 3 groups were collected for a total of 77 days and used to quantify the amount of viral RNAs in feces and serum samples by an HEV-specific RT-qPCR (56). We found that fecal virus shedding in the LR_Y1320H-infected rabbits occurred much earlier with higher viral RNA loads during the acute stage of HEV-3ra infection before 24 days postinoculation (dpi), and the fecal viral RNA loads were significantly higher at 14 dpi in LR_Y1320H-infected rabbits than that of animals infected with LR_WT (*P = *0.008) (Fig. 7B). Similarly, the serum viral RNA loads in LR_Y1320H-infected rabbits were numerically higher during the acute stage of HEV-3ra infection before 21 dpi, although the difference is not statistically significant (Fig. 7C). Therefore, the Y1320H mutation enhanced virus replication during the acute stage of HEV-3ra infection in rabbits, which is consistent with our *in vitro* results showing an enhanced viral replication of Y1320H compared to the LR_WT (Fig. 3D and E and Fig. 4C). Interestingly, after 24 dpi (feces) or 21 dpi (blood), the viral RNA loads in both feces and blood of LR_Y1320H-infected rabbits were numerically lower compared to those of LR_WT-infected rabbits until the end of the animal study at 77 dpi (Fig. 7B and C), although the difference is not statistically significant. It should be noted that the viral RNA loads in feces and blood of both LR_WT- and LR_Y1320H-infected rabbits were generally low and lasted for at least 77 dpi, indicating both LR_WT and LR_Y1320H induced persistent HEV-3ra infection in rabbits, which corroborates with an earlier report that the LR_WT caused persistent infection (27).

Seroconversion to IgG anti-HEV-3ra started at 21 dpi in both LR_WT- and LR_Y1320H-infected rabbits (Fig. 7D), concomitant with a rapid decrease of serum viral RNA loads in LR_WT-infected rabbits (28 dpi onward) and in LR_Y1320H-infected rabbits (21 dpi onward) (Fig. 7C). Notably, the anti-HEV-3ra IgG antibody titers in LR Y1320H-infected rabbits were numerically higher than those of LR_WT-infected rabbits (Fig. 7D), although the differences were not statistically significant. The anti-HEV IgG antibody titers were maintained in both LR_WT- and LR_Y1320H-infected rabbits until the end of the study at 77 dpi. As expected, all rabbits from the negative control group (PBS) remained seronegative throughout the study (Fig. 7D).

The viruses recovered from rabbits inoculated with LR_Y1320H were sequenced to verify the *in vivo* stabilities of the introduced mutation in the viral RdRp region. Sequence analyses revealed that the Y1320H mutation was maintained in the viruses recovered from feces, liver, and spleen collected at necropsy at 77 dpi, indicating that the Y1320H mutation is genetically stable in infected rabbits.

## DISCUSSION

Zoonotic HEV-3 infections have recently received considerable attention since they are associated with CHE in immunocompromised patients, particularly in solid-organ transplant recipients (10, 58), and with a number of neurological sequelae (12, 59). Although there is currently no specific therapy for chronic HEV-3 infections, abundant evidence has shown that RBV as monotherapy is effective in the treatment of CHE (11, 15). However, owing to the mutagenic effect of RBV on viruses, several amino acid mutations, including Y1320H, K1383N, D1384G, K1398R, V1479I, Y1587F, and G1634R, in the RdRp region of HEV-3 have reportedly occurred during RBV treatment in CHE patients (18, 20, 54). Of note, the Y1320H/K1383N/G1634R/triple mutant is associated with RBV resistance in a clinical case, and *in vitro* studies showed that Y1320H and G1634R enhance HEV replication, whereas K1383N decreases HEV replication but increases RBV susceptibility, thereby contradicting the clinical observation (21). More recently, additional amino acid substitutions (P25S, G38S, A64T, G71R, P79S, S95P, V245I, and T324S) in HEV-3 ORF2 protein are reportedly associated with a sustained viral response despite RBV therapy, and the P79S mutant impaired antibody-mediated neutralization of HEV-3, thus potentially acting as an immune decoy (20, 22). Nonetheless, due to the lack of a tractable and relevant animal model for chronic HEV-3 infection, the mechanisms of action or clinical relevance of these RBV treatment failure-associated mutations in HEV-3 replication and pathogenesis are largely unknown.

Rabbits are natural reservoirs of variant strains of HEV (25). Since the rabbit HEV variants and human HEV-3 phylogenetically cluster together, the ICTV has assigned rabbit HEV within the same genotype as human HEV-3, termed HEV-3ra (6). In our *in silico* analyses, we showed that HEV-3ra is evolutionarily closely related to human HEV-3, and HEV-3ra from rabbits has a significantly high level of sequence homology with human HEV-3, especially in the RdRp region (Fig. 1). Therefore, the HEV-3ra along with its cognate host, the rabbit, may potentially serve as a valuable and relevant model to elucidate the underlying mechanisms of RBV treatment failure-associated HEV-3 RdRp mutations identified in CHE patients. Furthermore, it has been shown that the HEV-3ra causes persistent infection and extrahepatic manifestations in rabbits, which could potentially serve as a much-needed smaller animal model to study the mechanism of chronic HEV-3 pathogenesis (27–31, 35–38).

By utilizing a rabbit HEV-3ra infectious clone, which is derived from an HEV-3ra LR strain and reportedly induces persistent HEV infection in rabbits (Fig. 1) (27), we determined the functional impact of the three most notable RBV treatment failure-associated HEV-3 RdRp mutations (Y1320H, K1383N, and G1634R) on replication and RBV antiviral sensitivity of the model rabbit HEV-3ra virus. We demonstrated that the Y1320H replicated and/or assembled much more efficiently than the WT in the Huh7-S10-3 liver cells, while the K1383N significantly impaired HEV-3ra replication. Neither K1634G nor K1634R significantly affected HEV-3ra replication compared to the WT. The K1634G replicated at a slightly reduced level and the K1634R at a slightly enhanced level (Fig. 3). Moreover, we constructed an HEV-3ra indicator replicon with the GLuc reporter system and used it to further validate our results through comparative mutational analyses. The results from the GLuc reporter system are consistent with those obtained with the infectious clone system (Fig. 4), suggesting that our results from this study are reproducible in two different systems. Surprisingly, a combinational double mutant containing the Y1320H/K1383N double mutations rescued the K1383N-associated replication defects. Furthermore, we demonstrated that the replication levels of HEV-3ra WT and mutants were significantly decreased, particularly for the Y1320H/K1383N mutant, when the cells were treated with RBV, indicating that the K1383N increased the RBV sensitivity of rabbit HEV-3ra (Fig. 5). Taken together, our data on the effect of three RBV treatment failure-associated HEV-3 RdRp mutations using the rabbit HEV-3ra LR model system is highly comparable with *in vitro* data obtained with human HEV-3 (21), indicating that rabbit HEV-3ra LR could serve as a relevant and useful model for studying the replication and antiviral sensitivity of RBV treatment failure-associated HEV-3 RdRp mutations. A major advantage of rabbit HEV-3ra over human HEV-3 is that the mutations in HEV-3ra can be readily tested with a homologous animal model since HEV-3ra naturally infects rabbits and causes persistent infection. Nevertheless, it should be noted that the rabbit HEV-3ra LR grows less efficiently in cultured cells than human HEV-3 Kernow-C1/p6 (21, 22, 50, 60–62).

There is a discrepancy in amino acid preference at position 1634 between HEV-3 from humans and pigs (G1634) and HEV-3ra from rabbits (K1634); moreover, whether R1634 merely occurs in CHE patients remains to be determined (19). To better understand the potential mechanism of these RdRp mutations in RBV resistance, we performed physicochemical analyses of the above-described three RBV treatment failure-associated HEV-3 RdRp mutations. Our results showed that the positively charged amino acids at positions 1320 and 1634 are preferable to hydrophobic amino acids for HEV-3 replication *in vitro*, but to what extent the biochemical properties of these distinctive amino acids affect HEV-3 replication remains an open question. Of note, the K1383N occurred in all four CHE patients with RBV treatment failure, and the selection of K1383N is reversible after the cessation of RBV treatment (20, 21). On the contrary, a retrospective multicenter study reported that the predominant RdRp mutations in HEV-3, e.g., K1383N, do not affect the viral clearance rate (54). We showed that the K1383 is located at motif I of HEV RdRp region, and structural prediction revealed that substitution of amino acid at position 1383 would alter the central three-dimensional structure of HEV RdRp (Fig. 6), which may explain the significant reduction of HEV-3 replication with the K1383N mutation. Indeed, the K1383 is highly conserved across eight different HEV genotypes, but the N1383 has only been observed in minor HEV-3 genomes of CHE patients (Fig. 2). Nonetheless, the exact mechanism of the K1383N mutation occurrence during chronic HEV-3 infection and RBV treatment remains elusive.

Since the Y1320H mutation had the most significant enhancement on the replication of HEV-3ra in our *in vitro* study, we proceeded to take advantage of the unique homologous rabbit model system of HEV-3ra to investigate whether the Y1320H mutation has similar impacts on virus replication *in vivo*. We found that fecal virus shedding in the HEV-3ra LR_Y1320H-infected rabbits occurred much earlier with higher viral RNA loads during the acute stage of virus infection before 24 dpi, and the fecal viral RNA loads were significantly higher at 14 dpi in LR_Y1320H-infected rabbits than those in rabbits infected with LR_WT (Fig. 7). Similarly, the serum viral RNA loads in LR_Y1320H-infected rabbits were numerically higher during the acute stage of virus infection before 21 dpi. Therefore, the Y1320H mutation appears to enhance virus replication during the acute stage of HEV-3ra infection in rabbits, which corroborated with our *in vitro* results showing an enhanced viral replication of LR_Y1320H compared to that of the LR_WT. However, after 21 dpi (blood) or 24 dpi (feces) until the end of the study, the viral RNA loads in both feces and blood of LR_Y1320H-infected rabbits were numerically lower compared to those of the LR_WT-infected rabbits, although these differences were not statistically significant. Seroconversion to IgG anti-HEV-3ra occurred at 21 dpi with numerically higher titers in LR_Y1320H-infected rabbits than in LR_WT-infected rabbits. The appearance of anti-HEV IgG antibodies was concomitant with a rapid decrease of serum viral RNA loads in LR_WT-infected rabbits (starting 28 dpi) and in LR_Y1320H-infected rabbits (starting 21 dpi). The heightened humoral immune response in the LR_Y1320H-infected rabbits would explain the decreased viral loads in infected animals after seroconversion. However, it is likely that the neutralizing antibodies in the infected rabbits failed to completely neutralize and clear the virus in rabbits, perhaps due to the presence of the quasi-enveloped form of viruses (9, 63), thus leading to the observed persistent infection. It should be noted that the Y1320 is evolutionarily conserved in nature among eight different HEV genotypes including rabbit HEV-3ra. Similar to the N1383, the H1320 merely occurred in a few HEV-3 genomes in CHE patients (Fig. 2), which may explain the less efficient *in vivo* replication of the LR_Y1320H than LR_WT during the late stage of HEV-3ra infection in rabbits (Fig. 7). Whether H1320 exists in natural strains of HEV-3 remains unknown.

Our *in vivo* results showed persistent HEV-3ra infection in rabbits, which is consistent with previous studies (27–31) and, therefore, may offer an opportunity in the future to study prolonged HEV-3 infection and its progression to chronicity. Despite this, whether persistent HEV-3 infection in rabbits could be recognized as a chronic infection, how to clearly define the measurable parameters of chronic HEV-3ra infection in rabbits and how the measurements correlate to chronic human HEV-3 infection remain to be determined in the future. Moreover, given the fact that HEV-3ra can persist in rabbits, it would be interesting, in future studies, to determine if RBV resistance mutations can develop in this unique rabbit HEV-3ra infection animal model when treated with RBV.

In conclusion, in this study, we demonstrated that rabbit HEV-3ra is evolutionarily closely related to human HEV-3. Thus, along with its cognate host (rabbit), HEV-3ra is a relevant and useful model to investigate the impact of RBV treatment failure-associated RdRp mutations of human HEV-3 on *in vitro* replication of HEV-3ra, as demonstrated by highly comparable *in vitro* results derived from human HEV-3. Importantly, we further revealed that the RBV treatment failure-associated Y1320H mutation significantly increased HEV-3ra replication efficiency *in vitro* and also enhanced HEV-3ra replication during the acute stage of virus infection at 14 dpi in rabbits. The appearance of anti-HEV-3ra IgG antibodies in infected rabbits coincided with a decreased virus replication, indicating that humoral immune response partially neutralized but did not clear virus infection in rabbits since the infected animals developed persistent HEV-3ra infections. These results collectively reveal that the HEV-3ra and its infection in rabbits are a relevant and useful model for studying the functional impact of RBV treatment failure-associated HEV-3 RdRp mutations identified in clinical cases.

## MATERIALS AND METHODS

### Ethics statement.

All experiments with rabbits were performed in strict accordance with the recommendations in the Guide for the Care and Use of Laboratory Animals of the American Veterinary Medical Association and the National Institutes of Health. The protocols were approved by the Institutional Animal Care and Use Committee (IACUC) at Virginia Tech (approval no. 21-252-CVM).

### Phylogenetic and sequence analyses.

Evolutionary analyses of rabbit HEV-3ra were conducted in Molecular Evolutionary Genetics Analysis (MEGA) (64) software for macOS version 11.0.11 with 1,000 bootstrap reiterations. A neighbor-joining tree of maximum likelihood distances was generated for the genus *Paslahepevirus*. The general time reversible (GTR) + gamma distributed (G) + invariable sites (I) nucleotide substitution model with the lowest Bayesian information criterion (BIC) score was selected for rabbit HEV-3ra based on the find best-substitution model (ML). Representative viral genomes of the eight HEV genotypes were included according to the proposed reference sequences for subtypes of HEV (genus *Paslahepevirus*) (6). In total, 52 rabbit HEV-3ra genomes were available and downloaded in the GenBank database (retrieved as of October 2022). Complete genomes are aligned using the multiple alignment using fast Fourier transform (MAFFT) (65) algorithm in Geneious Prime software version 2022.2.2.

A total of 1,018 complete viral genomes of eight HEV genotypes and the rabbit HEV-3ra within the genus *Paslahepevirus* (82 genomes for HEV-1, 2 genomes for HEV-2, 644 genomes for HEV-3, 52 genomes for HEV-3ra, 225 genomes for HEV-4, 2 genomes for HEV-5, 2 genomes for HEV-6, 3 genomes for HEV-7, and 6 genomes for HEV-8) analyzed in this study were downloaded in the GenBank database (retrieved as of October 2022). Genomic sequences from each genotype were aligned using the MAFFT algorithm in Geneious Prime software version 2022.2.2. Nucleotide and amino acid numbering are according to the HEV prototype strain from Burma (GenBank accession no. M73218) (6). Locations of functional domains and motifs within HEV ORF1 are according to the Burma strain (66).

### Rabbit HEV-3ra infectious clone and indicator replicon.

The rabbit HEV-3ra infectious clone pUC57-T7RHEV-LR (designated LR), which was derived from the HEV-3ra LR strain (GenBank accession no. LC484431), was kindly provided by Tian-Cheng Li (National Institute of Infectious Disease, Tokyo, Japan). The LR strain has been shown to induce persistent HEV-3ra infection in rabbits (27). To generate *Gaussia* luciferase replicon pUC57-T7RHEV-LR-Gluc (designated LRG), primers LR_Spel_FW and LR_ORF1Gluc_RV were used to amplify fragment A1 and primers LR_ORF2Gluc_FW and LR_EcoNI_RV were used to amplify fragment A2 with pUC57-T7RHEV-LR as the template, and primers Gluc_FW and Gluc_RV were used to amplify fragment Gluc with Kernow-C1 p6Gluc as the template (50). Three fragments were amplified with overlapping PCR with primers LR_Spel_FW and LR_EcoNI_RV and subsequently assembled into the pUC57-T7RHEV-LR vector (SpeI and EcoNI digested) (see Fig. S1 in the supplemental material). All primers used to construct the pUC57-T7RHEV-LR-Gluc are listed in Table S2 in the supplemental material.

### Site-directed mutagenesis and plasmid preparation.

Using rabbit HEV-3ra infectious clone LR or indicator replicon LRG as the backbone, the HEV-3ra mutants containing a single-site mutation, Y1320H, K1383N, K1634G, or K1634R, were constructed with the GeneArt site-directed mutagenesis system (Invitrogen-Thermo Fischer, Waltham, MA, USA) according to the manufacturer’s instructions. The LRG double mutant Y1320H/K1383N was constructed with GeneArt site-directed mutagenesis PLUS kit (Thermo Scientific, Waltham, MA, USA). As recommended for the mutagenesis system, PCRs were performed with AccuPrime Pfx DNA polymerase (Invitrogen-Thermo Scientific, Waltham, MA, USA). All primers used in this study for viral genomic sequencing and introduction of mutations are commercially synthesized (Integrated DNA Technologies, Coralville, IA, USA) and listed in Table S2.

One Shot MAX Efficiency DH5α-T1R competent cells (Invitrogen-Thermo Fischer, Waltham, MA, USA) were transformed with each of the HEV-3ra constructs containing specific mutation(s). The transformed cells were cultured in 50 mL lysogeny broth medium with ampicillin, and plasmid midipreps were performed with Qiagen Plasmid *Plus* midikit (Qiagen, Germantown, MD, USA). The entire viral genome in each of the HEV-3ra constructs was confirmed through Sanger sequencing to ensure that no additional nucleotide substitution had been introduced.

### Cell culture.

The Huh7-S10-3 cells are a subclone of Huh7 human hepatocarcinoma cells, kindly provided by Suzanne U. Emerson (NIAID, NIH, Bethesda, MD). The Huh7-S10-3 cells were cultured in Dulbecco’s minimal essential medium (DMEM) (Gibco-Thermo Fisher, Waltham, MA, USA) supplemented with 10% fetal bovine serum (FBS) (Atlanta Biologicals-R&D Systems, Minneapolis, MN, USA), 2 mM l-glutamine, 100 IU/mL antibacterial-antimycotic (Gibco-Thermo Fisher, Waltham, MA, USA), and 1% minimal essential amino acids (Gibco-Thermo Fisher, Waltham, MA, USA). Cells were kept at 37°C in a 5% (vol/vol) CO_2_ incubator.

### *In vitro* transcription and transfection.

The plasmids were linearized at the C terminus using the restriction enzyme XbaI (New England Biolabs, Ipswich, MA, USA). The linearized plasmid DNA was purified and used as the template for *in vitro* transcription of capped RNA with the mMESSAGE mMACHINE T7 ULTRA transcription kit (Thermo Fischer, Waltham, MA, USA). Viral RNA was transfected into Huh7-S10-3 cells with DMRIE-C transfection reagent (Thermo Fischer, Waltham, MA, USA) following the manufacturer’s instructions. Transfected Huh7-S10-3 cells were incubated at 34.5°C for 5 h. Subsequently, the transfection mixture was replaced with culture medium containing 10% FBS, and the incubation was continued at 34.5°C until further process (50).

### Real-time reverse transcription-quantitative PCR for quantification of rabbit HEV-3ra RNA.

To remove the residual input RNAs in cell culture from transfection, RNase A/T1 mix (Thermo Scientific, Waltham, MA, USA) was added to the cell culture supernatant according to the instructions. Extracellular viral RNA was extracted with QIAamp viral RNA minikit (Qiagen, Germantown, MD, USA). The HEV RNA loads were quantified using an established HEV-specific one-step RT-qPCR protocol with a SensiFAST Probe No-ROX one-step kit (Thomas Scientific, Swedesboro, NJ, USA) as described previously (56). The RT-qPCRs were performed on a CFX96 real-time system C1000 thermal cycler (Bio-Rad, Hercules, CA, USA). A standard curve was generated using *in vitro* transcribed viral RNA. Ten-fold serial dilutions of viral RNA, ranging from 1 × 10^7^ copies/μL to 1 × 10^2^ copies/μL, were used in each run. The viral genomic copy numbers were determined by interpolation of the standard curve and calculated as copies per milliliter of cell culture media or serum or copies per gram of feces (56).

### Luciferase assay and antiviral assay.

Seven days after transfection with rabbit HEV-3ra LRGluc indicator replicon, the cell culture medium (100 μL) was removed from a 96-well plate for analysis of extracellular luciferase expression activity. Cell culture media were collected at different time points to monitor the kinetic changes in luciferase expression activities. Luciferase activity was measured with the Pierce Gaussia Luciferase Glow assay kit (Thermo Scientific, Waltham, MA, USA). Briefly, 20 μL/well cell culture medium or cell lysate was added to each well of a black, opaque 96-well plate, followed by the addition of 50 μL Gaussia substrate. After 10 min incubation for signal stabilization, the luminescence was detected and recorded using a GloMax Discover microplate reader (Promega, Madison, WI, USA). Each LRG mutant replicon experiment was performed in quadruplicate. Luminescence-based antiviral assay for HEV was performed with ribavirin (Sigma-Aldrich, St. Louis, MO, USA) at a concentration of 1 μM or 10 μM following an established protocol (59).

### Immunofluorescence assay.

Huh7-S10-3 cells transfected with viral RNA transcripts were fixed at 7 days posttransfection with cold 100% methanol and blocked with 5% bovine serum albumin (BSA). Subsequently, the cells were probed using a rabbit anti-HEV ORF2 polyclonal antibody (1:50) (67), followed by incubation with a secondary antibody, goat anti-rabbit Alexa Fluor 488 (1:1,000) (Thermo Scientific, Waltham, MA, USA). The nuclei were stained with 4′,6-diamidino-2-phenylindole (DAPI). The stained cells were visualized under the imaging multi-mode reader BioTek Cytation 5 microscope (Fisher Scientific, Waltham, MA, USA). The numbers of HEV-3ra ORF2-positive cells were counted and quantified using ImageJ2/FIJI software version 2.9.0/1.53t (50, 61).

### Physicochemical and structural analyses.

The physicochemical properties and structures of relevant proteinogenic amino acids in this study are illustrated and adapted from https://commons.wikimedia.org/wiki/File:Proteinogenic_Amino_Acid_Table.png created by Thomas Ryckmans (2022). Structural comparisons were conducted of RdRp of wild-type rabbit HEV-3ra LR or that with each single RBV treatment failure-associated HEV-3 RdRp mutation. The three-dimensional structure of RdRp is predicted with AlphaFold (68) and visualized and annotated in Geneious Prime software version 2022.2.2.

### Experimental infection of rabbits with HEV-3ra wild-type and Y1320H mutant viruses.

The rabbit HEV-3ra LR wild-type (LR_WT) and Y1320H mutant (LR_Y1320H) were propagated in Huh7-S10-3 cells to generate infectious virus stocks. The cell culture supernatant obtained from Nunc EasYFlask 75 cm^2^ (Thermo Scientific, Waltham, MA, USA) was passed through 0.45-μm Nalgene sterile syringe filters (Thermo Scientific, Waltham, MA, USA) to remove cell debris. The genomic RNA copy number for the virus inocula of LR_WT and LR_Y1320H were measured and unified with 2.6 × 10^7^ copies/mL before and 2.1 × 10^6^ copies/mL after RNase A/T1 treatment. To further determine the infectious virus titer of each inoculum, we infected the HepG2 liver cells with LR_WT and LR_Y1320H and determined the 50% tissue culture infectious dose (TCID_50_) using immunofluorescence assays (IFA). Since HEV is a nonlytic virus and no cytopathic effect can be visualized in infected cells, we therefore determine the TCID_50_ by microscopically counting HEV-3ra-positive foci. The TCID_50_ determination for the HEV inoculum has been described in detail from our previous study (59). A total of 15 specific-pathogen-free (SPF) 6-month-old female New Zealand White rabbits, which tested negative for anti-HEV IgG and IgM antibodies and for rabbit HEV RNA, were divided into three groups of 5 animals each. One group of rabbits was intravenously inoculated via the ear vein with LR_WT (~3 × 10^3^ TCID_50_ infectious doses per rabbit), another group was similarly inoculated with LR_Y1320H (~3 × 10^3^ TCID_50_ infectious doses per rabbit), and the remaining group of animals was similarly inoculated with PBS as negative control. This animal study is approved by the Virginia Tech Institutional Animal Care and Use Committee (IACUC).

Fecal samples were collected prior to inoculation and twice weekly, and serum samples were obtained weekly from each of the inoculated rabbits. Fecal materials were diluted with PBS to prepare a 10% (wt/vol) fecal suspension. The fecal suspension was then clarified by centrifugation at 10,000 × *g* for 15 min and filtered through a 0.45-μm membrane. Viral RNAs from rabbit fecal and serum samples were extracted using TRI Reagent and Quick-RNA viral kit (Zyno Research, Irvine, CA, USA), respectively. The fecal and serum samples were used for the quantification of HEV RNA loads by RT-qPCR, and the serum samples were also used for the detection of anti-HEV IgG antibodies by enzyme-linked immunosorbent assay (ELISA). All rabbits were necropsied at 11 weeks (77 days) postinoculation.

### Statistical analysis.

All statistical tests were performed in GraphPad Prism for macOS software version 9.4.1. Comparisons among three or more experimental groups in cell culture were performed with one-way analysis of variance (ANOVA) with Tukey’s multiple comparison test. To evaluate the difference between HEV-3ra WT and Y1320H in inoculated rabbits at each time point, a two-sided, unpaired, multiple Student's *t* test without adjustments was performed. Differences were considered statistically significant when the *P* value was less than 0.05.

### Data availability.

The nucleotide sequence of the rabbit HEV RHEV-LR Gluc (LRG) indicator replicon generated in this study has been deposited into GenBank under accession no. OP887158.
