# Supplementary material for: Ribavirin Treatment Failure-Associated Mutation, Y1320H, in the RNA-Dependent RNA Polymerase of Genotype 3 Hepatitis E Virus (HEV) Enhances Virus Replication in a Rabbit HEV Infection Model
Source: mBio. 2023 Feb 21;14(2):e03372-22. doi: 10.1128/mbio.03372-22 (PMC10128057; doi:10.1128/mbio.03372-22)
Supplement: TABLE S1 [file mbio.03372-22-s0002.docx]

**Table S1.** Prevalence of different amino acid residues at positions of the three Ribavirin treatment failure-associated HEV-3 RdRp mutations for each of the eight HEV genotypes and rabbit HEV-3ra within the genus *Paslahepevirus*

| HEV Genotype | 1 | 2 | 3 | 3ra | 4 | 5 | 6 | 7 | 8 |
| --- | --- | --- | --- | --- | --- | --- | --- | --- | --- |
| No. of viral genomes | 82 | 2 | 644 | 52 | 225 | 2 | 2 | 3 | 6 |
| Y1320 | 82 (100)*^a^* | 2 (100) | 638 (99.06) | 52  (100) | 225 (100) | 2 (100) | 2 (100) | 3 (100) | 6 (100) |
| H1320 | 0 | 0 | 2 (0.31) | 0 | 0 | 0 | 0 | 0 | 0 |
| N1320 | 0 | 0 | 1 (0.16) | 0 | 0 | 0 | 0 | 0 | 0 |
| F1320 | 0 | 0 | 3 (0.47) | 0 | 0 | 0 | 0 | 0 | 0 |
|  |  |  |  |  |  |  |  |  |  |
| K1383 | 82 (100) | 2 (100) | 637 (98.91) | 52 (100) | 215 (99.56) | 2 (100) | 2 (100) | 3 (100) | 6 (100) |
| N1383 |  |  | 7 (1.09) |  |  |  |  |  |  |
| R1383 |  |  |  |  | 10 (0.44) |  |  |  |  |
|  |  |  |  |  |  |  |  |  |  |
| G1634 |  |  | 553 (85.87) |  | 1 (0.44) |  |  |  |  |
| R1634 |  | 1 (50) | 83 (12.89) | 4 (7.69) | 23 (10.22) | **2** (100) |  |  | 1 (16.67) |
| K1634 | 81 (98.78) | 1 (50) | 8 (1.24) | 48 (92.31) | 201 (89.34) |  | **2** (100) | **2** (100) | 5 (83.33) |
| Q1634 | 1 (1.22) |  |  |  |  |  |  |  |  |

*^a^*Number of viral genomes (% prevalence)
