# Supplementary material for: Ribavirin Treatment Failure-Associated Mutation, Y1320H, in the RNA-Dependent RNA Polymerase of Genotype 3 Hepatitis E Virus (HEV) Enhances Virus Replication in a Rabbit HEV Infection Model
Source: mBio. 2023 Feb 21;14(2):e03372-22. doi: 10.1128/mbio.03372-22 (PMC10128057; doi:10.1128/mbio.03372-22)
Supplement: TABLE S2 [file mbio.03372-22-s0003.docx]

**Table S2.** Primers used for rabbit HEV-3ra LR strain genomic sequencing, construction of viral mutants, and generation of LRGluc indicator replicon

| Primer ID*^a^* | Sequence*^b^* (5’-3’) | Application*^c^* |
| --- | --- | --- |
| LR_13_FW | AAGTGGTCGATGCCATGGAG | Genomic sequencing for LR/G |
| LR_585_FW | TATGCTGTGTTGCACCTCCC |  |
| LR_1342_FW | CTGCCGGCTTTCATTTGGAC |  |
| LR_2071_FW | TTTCCCCCGGTCATTTCTGG |  |
| LR_2821_FW | TAGTCCTTGACACGCCCTTG |  |
| LR_3597_FW | CTCATCCAGTCATCTCGGGC |  |
| LR_4313_FW | TCAGGGGATATCAGCCTGGA |  |
| LR_5066_FW | GGTGAGTCCTGGGCTTGTAC |  |
| LR_5832_FW | TGCAGTTGGTGGCTATGCAA |  |
| LR_6565_FW | AACCAACATGAGCAGGACCG |  |
| LR_pol_3525F_FW | ATTACAGTTCATGAGGCCCAGG |  |
| LR_pol_3653F_FW | GTGTGTTATTTTAGACGCCCCG |  |
| LR_pol_5267R_RV | ACGACGAACAGCAGCAAAATAG |  |
| LR_Y1320H_FW | CGTCGAACAAGGTTA**CAT**GAGGCCGCTCACT | Mutagenic for LR/G_Y1320H |
| LR_Y1320H_RV | AGTGAGCGGCCTCAT**GTA**ACCTTGTTCGACG |  |
| LR_K1383N_FW | AACTTTCTTCCAG**AAT**GACTGTAACAAGTTC | Mutagenic for LR/G_K1383N |
| LR_K1383N_RV | GAACTTGTTACAGTC**ATT**CTGGAAGAAAGTT |  |
| LR_K1634G_FW | TGTGATTTCCTGAG**AGG**GTTGACGAATGTTGC | Mutagenic for LR/G_K1634G |
| LR_K1634G_RV | GCAACATTCGTCAAC**CCT**CTCAGGAAATCACA |  |
| LR_K1634R_FW | GTGATTTCCTGAG**AAG**GTTGACGAATGTTGC | Mutagenic for LR/G_K1634R |
| LR_K1634R_RV | GCAACATTCGTCAAC**CTT**CTCAGGAAATCAC |  |
| LR_SpeI_FW | CAGTATCTGACAGTGTACTAGT | RHEV-LR-Gluc replicon construction |
| LR_EcoNI_RV | GCCCCAAAGCACCTGTGTAAGG |  |
| LR_ORF1Gluc_FW | TCAAGGGGGCCGGTGGTGACTAAGCCGCCAGTATAATCTCT |  |
| LR_ORF2Gluc_RV | AACAGAACTTTGACTCCCATGGTGAACCCATGGGCGATGC |  |
| Gluc_FW | ATGGGAGTCAAAGTTCTGTTTGC |  |
| Gluc_RV | GTCACCACCGGCCCCCTTGATCTTG |  |

*^a^*Forward primer designations end with “_FW”; reverse primer designations end with “_RV”.

*^b^*Bold and underlined nucleotides indicate mutagenesis and cloning purposes, respectively.

*^c^*LR/G stands for rabbit HEV-3ra infectious clone pUC57-T7RHEV-LR and luciferase replicon pUC57-T7RHEV-LR-Gluc.
