# Supplementary figures and images for: Ribavirin Treatment Failure-Associated Mutation, Y1320H, in the RNA-Dependent RNA Polymerase of Genotype 3 Hepatitis E Virus (HEV) Enhances Virus Replication in a Rabbit HEV Infection Model
Source: mBio. 2023 Feb 21;14(2):e03372-22. doi: 10.1128/mbio.03372-22 (PMC10128057; doi:10.1128/mbio.03372-22)

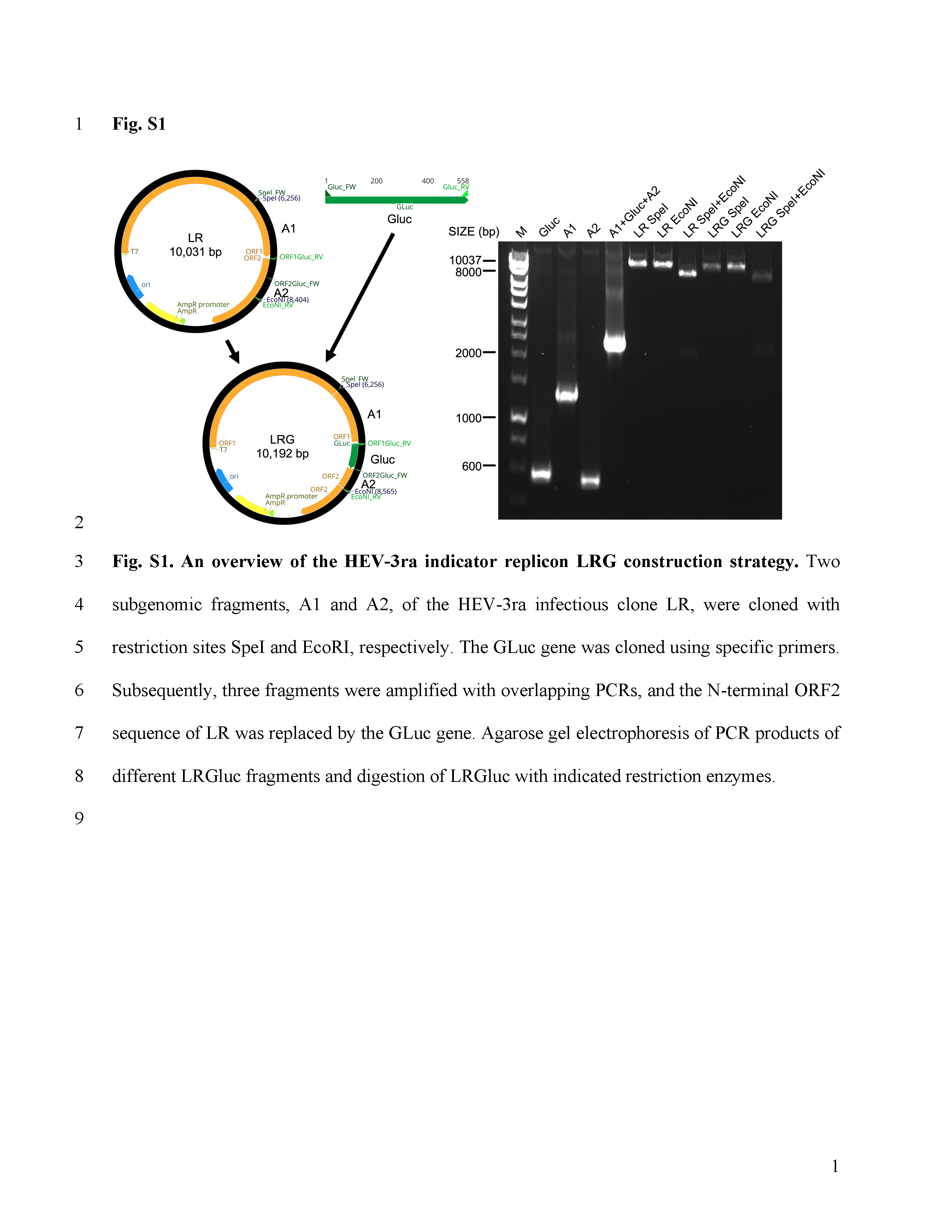

Supplement: FIG S1 [file mbio.03372-22-s0001.tif]
